# Neuralgia Facei

**Published:** 1859-07

**Authors:** J. P. H. Brown

**Affiliations:** Dentist, of Atlanta, Georgia


### ARTICLE V.

*Neuralgia Facei.* By J. P. H. Brown, Dentist, of Atlanta, Georgia.

In the treatment of some few diseases, the respective spheres of the dentist and the physician seem to approach so closely, that it is a difficult matter to define precisely the line of demarcation between them. Facial neuralgia occupies such a position:

In considering this subject it is not my intention to encroach upon the ground occupied by the practitioner of medicine, but only to call his attention to the claims of the dentist—to the great influence exerted by diseased and misplaced teeth in the production of this disease.

Neuralgia facei usually occurs in some of the branches of the *trigeminus*, or fifth pair of nerves, and is sometimes recognised by paroxysms of acute, lancinating pain, shooting from the region of the mouth, to the eyes, ears, cheeks, temples, and teeth. These paroxysms often return with great regularity at particular seasons of the year, as spring or fall; and are generally of a quotidian, tertian, or quartan character. This form of neuralgia has been termed *intermittent*, and is often *idiopathic*, and an attendant of malaria, or cold, or of a rheumatic or arthritic habit; or it may be *sympathetic*, and a result of disease in some organ, as the stomach, liver, uterus, etc.

The *local* form of facial neuralgia is caused by some affection of the dental apparatus. It may arise from a diseased tooth, a dead or exostosed fang, a crowded and misplaced condition of the teeth, ulceration of the gums, or from necrosis of the alveolar processes, or of the maxillary bones.

As one well established fact is worth a whole ton of theory, I will select the following cases from my note-book:

CASE I.—The patient, aged 23, was the wife of a practicing physician. She had been suffering—with some slight intermissions—with facial neuralgia for two months. Her husband had exhausted his skill and his remedies to no purpose. When I was called to see her, I found her whole system prostrated; and she was suffering with sharp, lancinating pains in her cheeks and temples. At times the pain would start in the inferior maxillary, opposite the roots of the molar teeth, and shoot to the neck; thence to the eyes and brows. Upon an examination of her mouth, I found the first and second molar upon the one side, and the first molar upon the other side of the lower jaw, all containing large nerve fillings. These teeth seemed to be in good condition, and looked rather well; but upon close inspection I found that they had, by an injudicious use of arsenious acid in the destruction of their nerves, not only become deprived of their vitality, but that their periosteal attachment to the jaw was also in a great measure destroyed. Hence they were dead structures, and acted as morbid irritants to the dental nerves. After stating my opinion of her case to her husband, he agreed with me in the diagnosis, and urged the extraction of the teeth, which operation I performed. Her neuralgia immediately left her.

Case II.—In December, 1856, a gentleman, aged about 25, called to consult me in reference to a severe neuralgia from which he was then suffering. He had been under the treatment of two of the best physicians in Western Maryland, but they gave him no relief. I found his teeth perfectly sound, and of rather good quality; and nothing about them that would lead one to suppose that they had anything to do with the matter. Seeing that the jaw was short in length, and the arch narrow and contracted; and that one of the inferior *dentes sapientia* was *erupted* but not the other. I came to the conclusion that the un-cut tooth was from want of room, misplaced in the jaw, and gave rise to the difficulty. After lancing the gum I discovered the wisdom tooth deeply imbedded, and lying in a horizontal position, between the fangs of the second molar and the *ramus* of the jaw. After considerable difficulty I succeeded in extracting it. There was an immediate subsidence of the neuralgic pains.

From the above cases, we may see the importance of paying strict attention to, and of closely examining the condition of the dental organs, particularly when we consider their nervous connexions and wide-spread sympathetic relations, before we attempt to prescribe our treatment for neuralgia; for, if we do not, we may often expect to be chagrined by finding our favorite remedies proving of no avail.

